# Supplementary material for: Strain concentration drives the anatomical distribution of injury in acute and chronic traumatic brain injury
Source: bioRxiv. 2024 May 23:2024.05.22.595352. Preprint. [Version 1] doi: 10.1101/2024.05.22.595352 (PMC11142169; doi:10.1101/2024.05.22.595352)
Supplement: Supplement 1 [file NIHPP2024.05.22.595352v1-supplement-1.pdf]

bioRxiv preprint doi: https://doi.org/10.1101/2024.05.22.595352; this version posted May 23, 2024. The copyright holder for this preprint (which was not certified by peer review) is the author/funder, who has granted bioRxiv a license to display the preprint in perpetuity. It is made available under aCC-BY-NC-ND 4.0 International license.

Supplemental Figure 1: Overlap and dissociation of high strain regions for 30, 50 and 70 Hz occipital actuation MRE data. Strain concentration defined at a threshold of T = 4 (FDR-corrected q < .05) for 30 Hz (copper), 50 Hz (green), and 70 Hz (yellow) and intersection (blue). Blue colored regions experience differentially high strain concentration across all three tested frequencies.

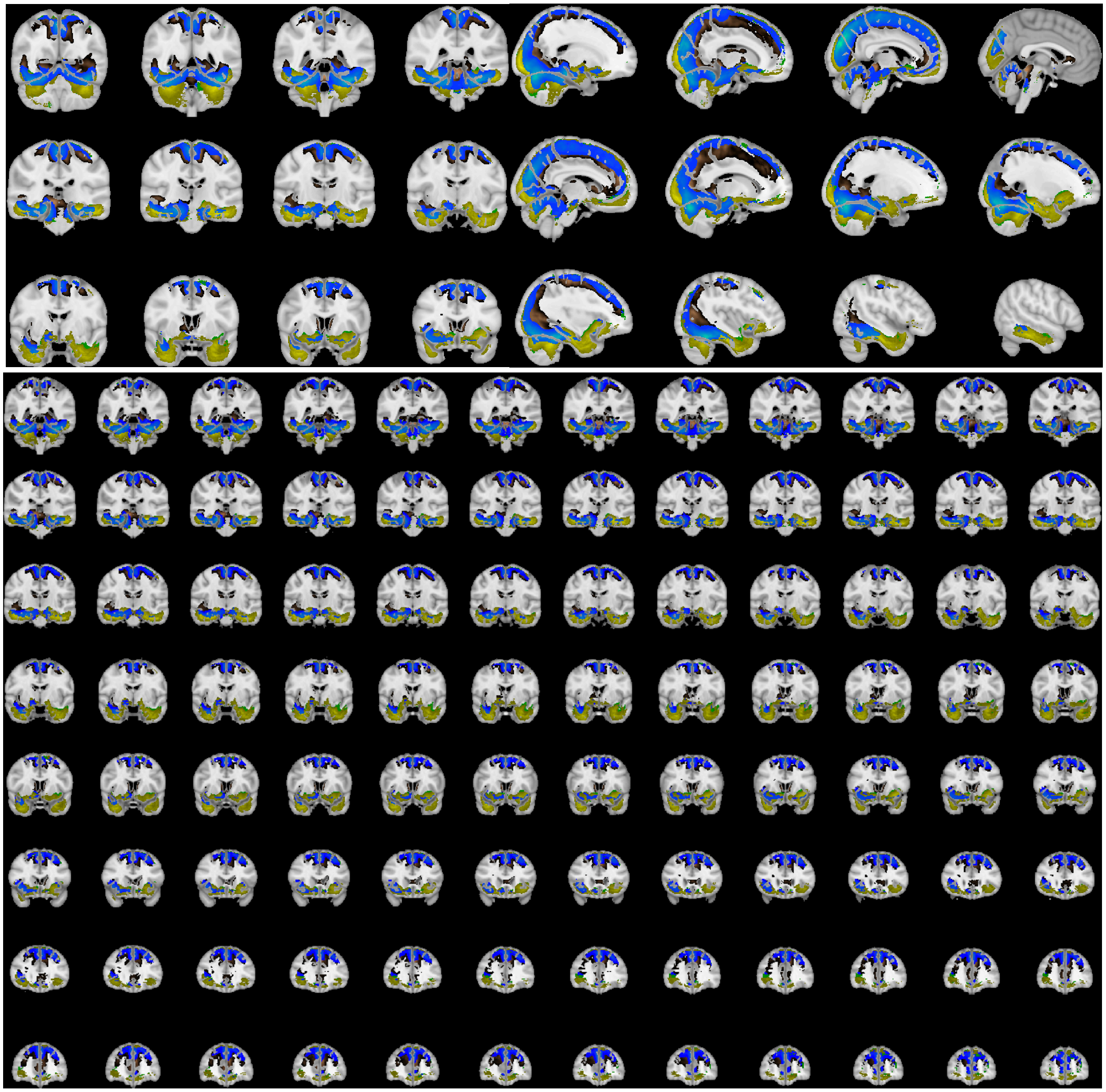

.
